# Enhancing Cardiac Rehabilitation Referral at Discharge Using EHR-Based Decision Support for Guideline-Eligible Patients

**DOI:** 10.1016/j.jaccas.2026.110346

**Published:** 2026-09-16

**Authors:** Mohanad Albayyaa, Shiva Gollapally Krishna, Qamar Othman Abdullah, Ibrahim Mortada, Riham Dheyab, Abdul Qadeer, Carolina Colli Cruz, Jesús Gibran Hernández-Pérez, Omer Abdelgadir, David S. Lopez, Hani Jneid, Bashar Al hemyari

**Affiliations:** a Department of Cardiovascular Medicine, University of Texas Medical Branch, Galveston, Texas, USA; b Department of Clinical Pharmacy, College of Pharmacy, Ashur University, Baghdad, Iraq; c Department of Pharmacy, Kelsey Seybold Clinic, Pearland, Texas, USA; d Peter O’Donnell School of Public Health, UT Southwestern Medical Center, Dallas, Texas, USA

**Keywords:** CABG, cardiac rehabilitation, heart failure, heart transplant, myocardial infarction, percutaneous coronary intervention, valve surgery, TAVR

## Abstract

**Background:**

Cardiac rehabilitation (CR) is a guideline-recommended therapy that improves outcomes in cardiovascular disease, yet referral at hospital discharge remains suboptimal.

**Project Rationale:**

Variability in discharge workflows and inconsistent use of standardized referrals contributed to missed CR referrals among eligible patients.

**Project Summary:**

We implemented an electronic health record (EHR)–based best practice advisory within the Epic EHR system to prompt CR referral or documentation of nonreferral reasons for guideline-eligible cardiovascular patients. A retrospective quality improvement study compared preimplementation (February 1, 2022–January 31, 2024) and postimplementation (February 1, 2024–January 31, 2026) cohorts (N = 21,294). Outcomes assessed included CR referral orders, documentation of referral activity, and CR-related activity. CR referral rates increased from 45.3% to 86.7% after best practice advisory implementation.

**Take-Home Messages:**

EHR-based decision support improved CR referral rates among guideline-eligible patients by integrating referral prompts into discharge workflows. Continued optimization of provider engagement and workflow integration may further improve referral completion.

## Graphical Abstract

**Figure:**
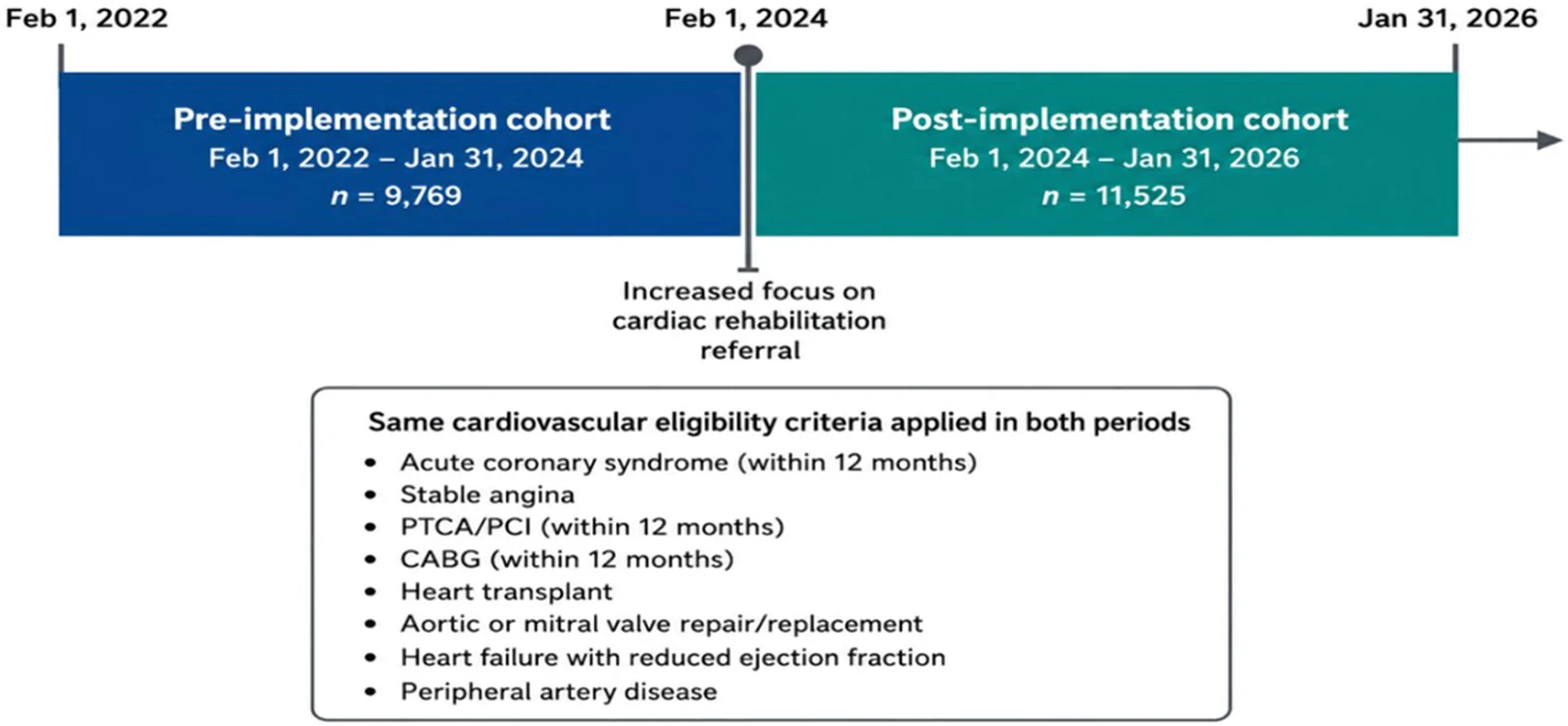

Cardiac rehabilitation (CR) is a cornerstone of secondary prevention in cardiovascular disease and is strongly recommended by contemporary clinical practice guidelines. Participation in CR is associated with reductions in all-cause mortality, cardiovascular mortality, and hospital readmissions, while improving functional capacity, quality of life, and adherence to evidence-based therapies among patients with cardiovascular disease.^1^, ^2^, ^3^ Despite these well-established benefits, CR remains substantially underused nationwide.

National studies consistently demonstrate low referral and participation rates among eligible patients following acute coronary syndromes, percutaneous coronary intervention, coronary artery bypass grafting, and other qualifying cardiovascular conditions.^2^^,^^3^ Referral practices often rely on individual provider discretion and workflow patterns, resulting in significant variability in referral rates across institutions and clinical teams.^4^^,^^5^ Consequently, many eligible patients are discharged without access to a proven secondary prevention intervention.

Previous implementation studies have identified system-level barriers, including competing discharge priorities, lack of standardized referral processes, and limited integration of referral workflows within the electronic health record (EHR).^5^^,^^6^ Automated referral strategies and EHR-based clinical decision support tools have demonstrated effectiveness in increasing CR referrals and improving adherence to guideline-directed care.^6^, ^7^, ^8^

At the University of Texas Medical Branch (UTMB Health), internal quality assessments identified low CR referral rates among guideline-eligible hospitalized cardiovascular patients. Analysis of existing workflows revealed inconsistent referral practices and the absence of standardized EHR prompts during discharge planning. To address these gaps, we implemented a best practice advisory (BPA) within the Epic EHR system designed to prompt CR referral or documentation of clinician-selected reasons for not placing a referral when applicable. This quality improvement (QI) initiative evaluated the impact of BPA implementation on cardiac rehabilitation referral practices, referral documentation, and workflow adherence.

## Project Rationale

The underuse of CR reflects a persistent implementation gap in cardiovascular care, despite strong evidence supporting its benefits and clear guideline recommendations. Prior studies have demonstrated that structured referral pathways, automatic referral systems, and electronic clinical decision support tools can significantly improve CR referral and participation rates. Nevertheless, adoption of these strategies remains inconsistent across health care systems, resulting in substantial variation in referral practices and missed opportunities for secondary prevention.

At UTMB Health, internal quality assessments identified low CR referral rates among hospitalized patients who met guideline-based eligibility criteria. Review of existing discharge workflows revealed reliance on individual provider practices and the absence of standardized EHR-based prompts to support CR referral decisions. These findings highlighted the need for a scalable, workflow-integrated intervention to promote consistent adherence to guideline-directed care.

We hypothesized that embedding a BPA within the Epic EHR at the point of discharge would standardize referral practices, reduce provider-level variability, and increase CR referrals among eligible cardiovascular patients. The objective of this QI initiative was to evaluate the impact of BPA implementation on cardiac rehabilitation referral rates, referral documentation, and adherence to guideline-directed referral practices.

## Project Description

We conducted a retrospective QI study using aggregated EHR data from Epic at a large academic medical center.

## Population

Patients were identified using guideline-based eligibility criteria for CR, as shown in Figure 1, including:

• Acute coronary syndrome within 12 months
• Stable angina
• Percutaneous coronary intervention or percutaneous transluminal coronary angioplasty within 12 months
• Coronary artery bypass grafting within 12 months
• Heart transplant
• Valvular repair or replacement
• Heart failure with reduced ejection fraction
• Peripheral artery disease

**Figure 1:**
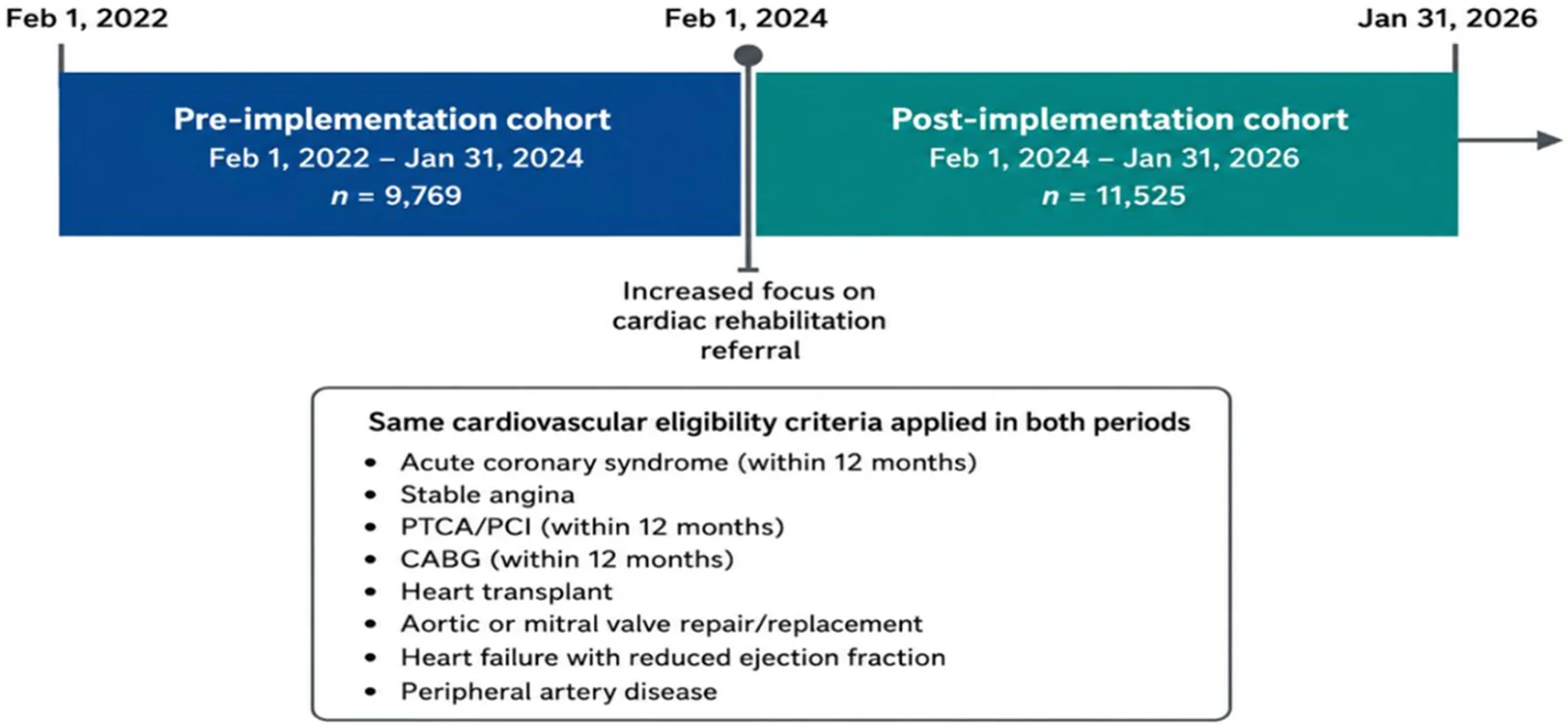
Study Design and Cohort Identification Timeline CABG = coronary artery bypass grafting; PCI = percutaneous coronary intervention; PTCA = percutaneous transluminal coronary angioplasty.

## Study Period

• Preimplementation: February 1, 2022 to January 31, 2024
• Postimplementation: February 1, 2024 to January 31, 2026

## Intervention

An Epic-based BPA was implemented as an electronic clinical decision support tool to improve adherence to guideline-directed CR referral practices. The BPA was triggered during the hospital discharge process for patients meeting predefined cardiovascular eligibility criteria for CR. When activated, the BPA required providers to either place a CR referral order or document a clinical reason for not placing a referral. Providers could acknowledge the BPA and either complete the referral workflow or document the reason that referral was not placed. This approach was designed to integrate CR referral into routine discharge workflows, promote consistent referral practices, and reduce provider-level variability in referral decisions. The intervention was developed and implemented through a multidisciplinary collaboration involving cardiology clinicians, nursing staff, QI personnel, Epic analysts, and clinical informatics specialists.

## Data Collection and Outcomes

Data were extracted retrospectively from the Epic EHR using SlicerDicer queries based on predefined cardiovascular eligibility criteria, as shown in Figure 1. Outcomes were assessed in both the preimplementation and postimplementation cohorts and included the following:

• CR referral orders and related documentation activity
• Documentation of clinician-selected reasons for nonreferral

CR-related activity was defined as the presence of a CR referral order, documentation related to CR referral, or CR-related encounters recorded within the EHR. CR-related activity included placement of a CR referral order, documentation of referral-related activity, or documentation of clinician-selected reasons for not placing a referral. Outcomes focused on evaluating changes in referral practices following implementation of the BPA, as shown in Figure 2.

**Figure 2:**
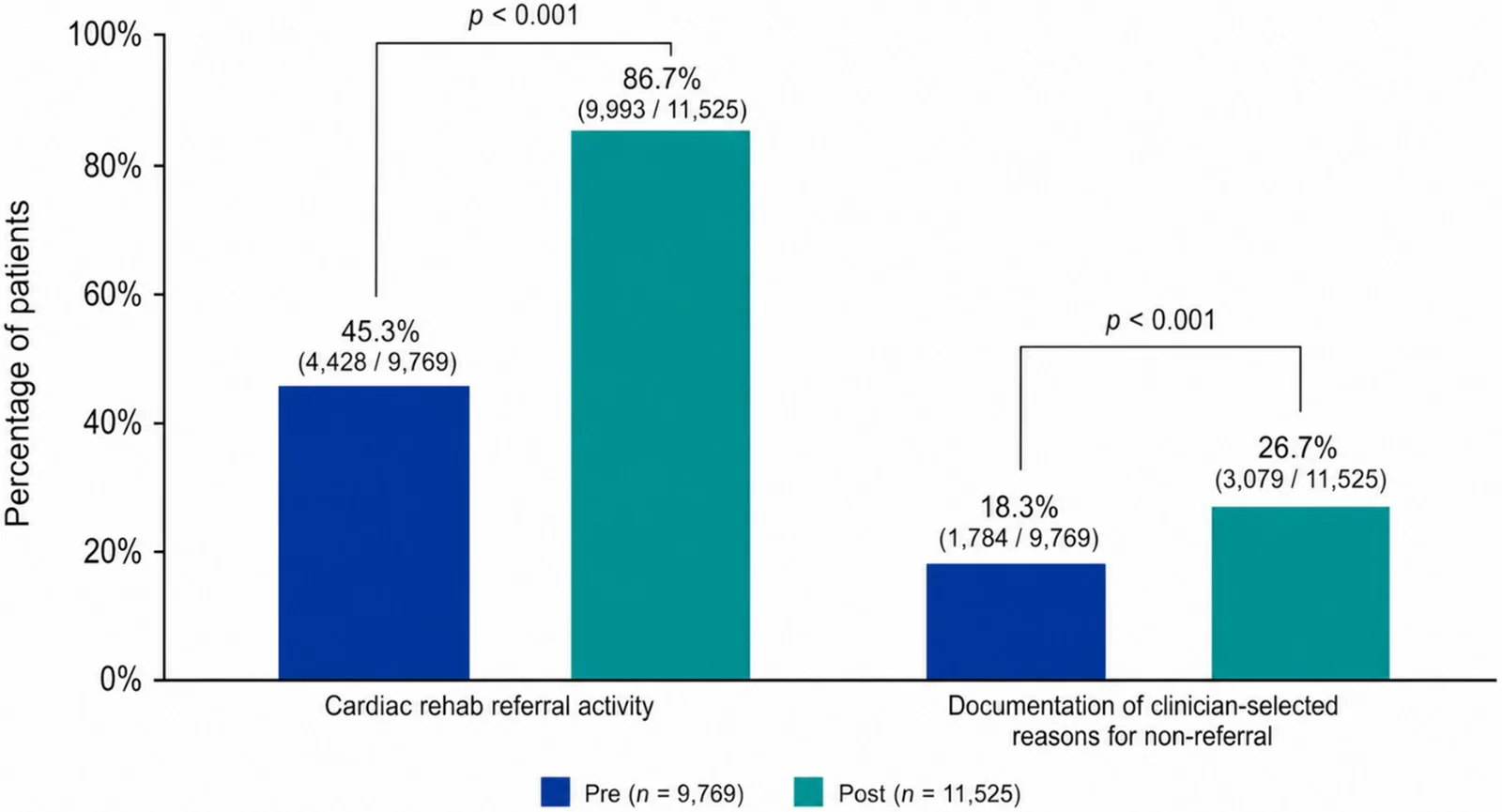
Cardiac Rehabilitation Referral Activity Before and After Best Practice Advisory Implementation

## Project Deliverables

Key project deliverables included the following:

• Integration of the CR BPA into Epic discharge workflows for guideline-eligible cardiovascular patients.
• Automated identification of CR-eligible patients using standardized EHR-based clinical criteria.
• Development and implementation of a structured CR referral order workflow embedded within the discharge process.
• Standardized documentation of clinician-selected reasons when cardiac rehabilitation referral was not placed.
• Creation of a reproducible EHR-based cohort identification and monitoring framework to support ongoing QI efforts and performance tracking.

## Outcome, Impact, and Future Directions

A total of 21,294 eligible patients were identified, including 9,769 in the preimplementation cohort and 11,525 in the postimplementation cohort. Following implementation of the Epic-based BPA, cardiac rehabilitation referral rates improved from 45.3% to 86.7%, with increased adherence to structured referral documentation requirements, reflecting improved adherence to guideline-directed referral practices and standardization of discharge workflows, and enhanced documentation of reasons for nonreferral (Figure 3). A comparison of the number of CR-related activities between preintervention and postintervention is shown in Figure 4. This intervention demonstrated improved standardization of discharge workflows and reduced variability in referral practices across providers. Future efforts will focus on sustaining long-term adherence, refining eligibility logic within the EHR, and expanding the intervention to additional discharge quality metrics.

**Figure 3:**
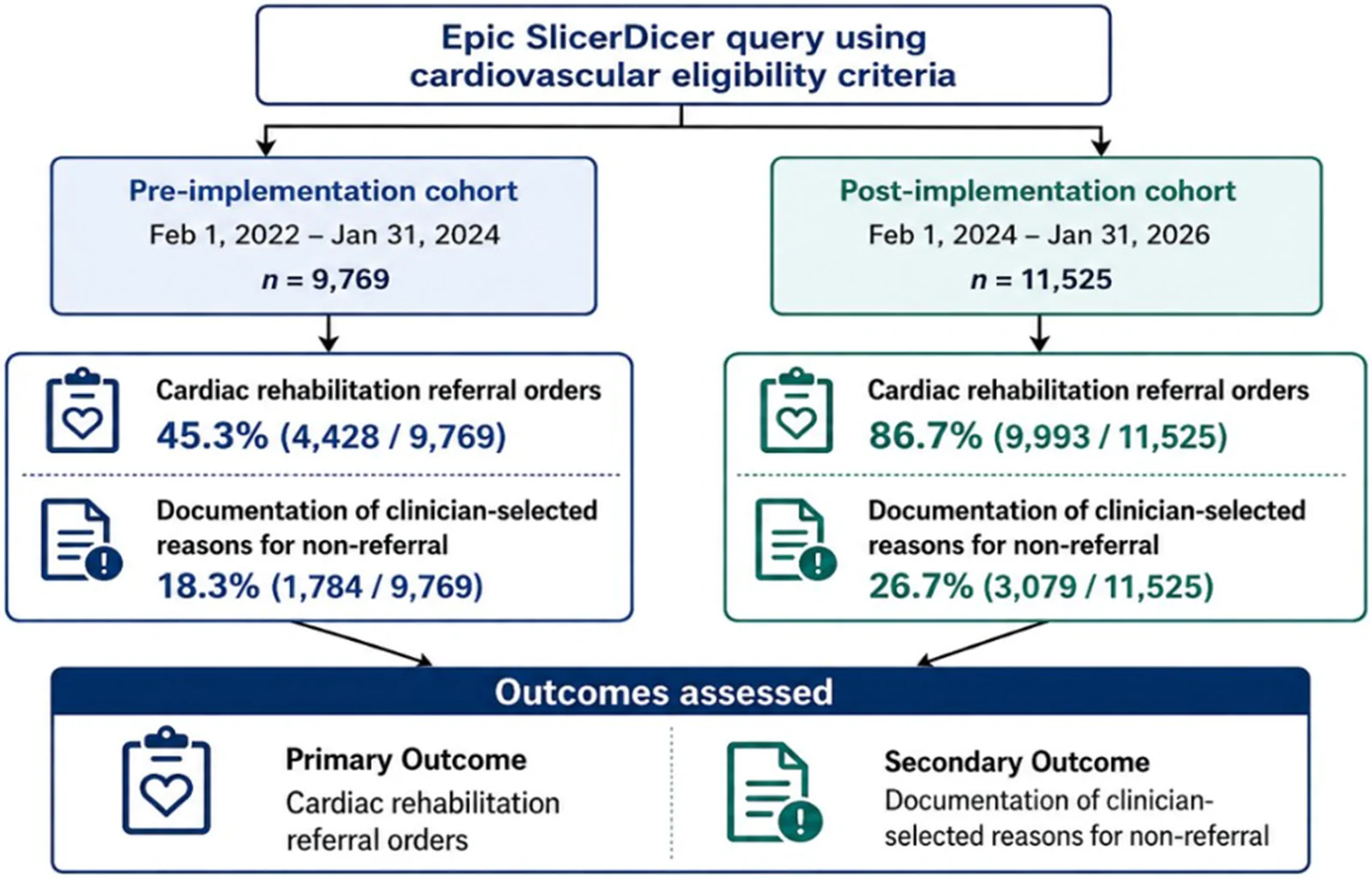
Flowchart of Cohort Selection and Outcomes

**Figure 4:**
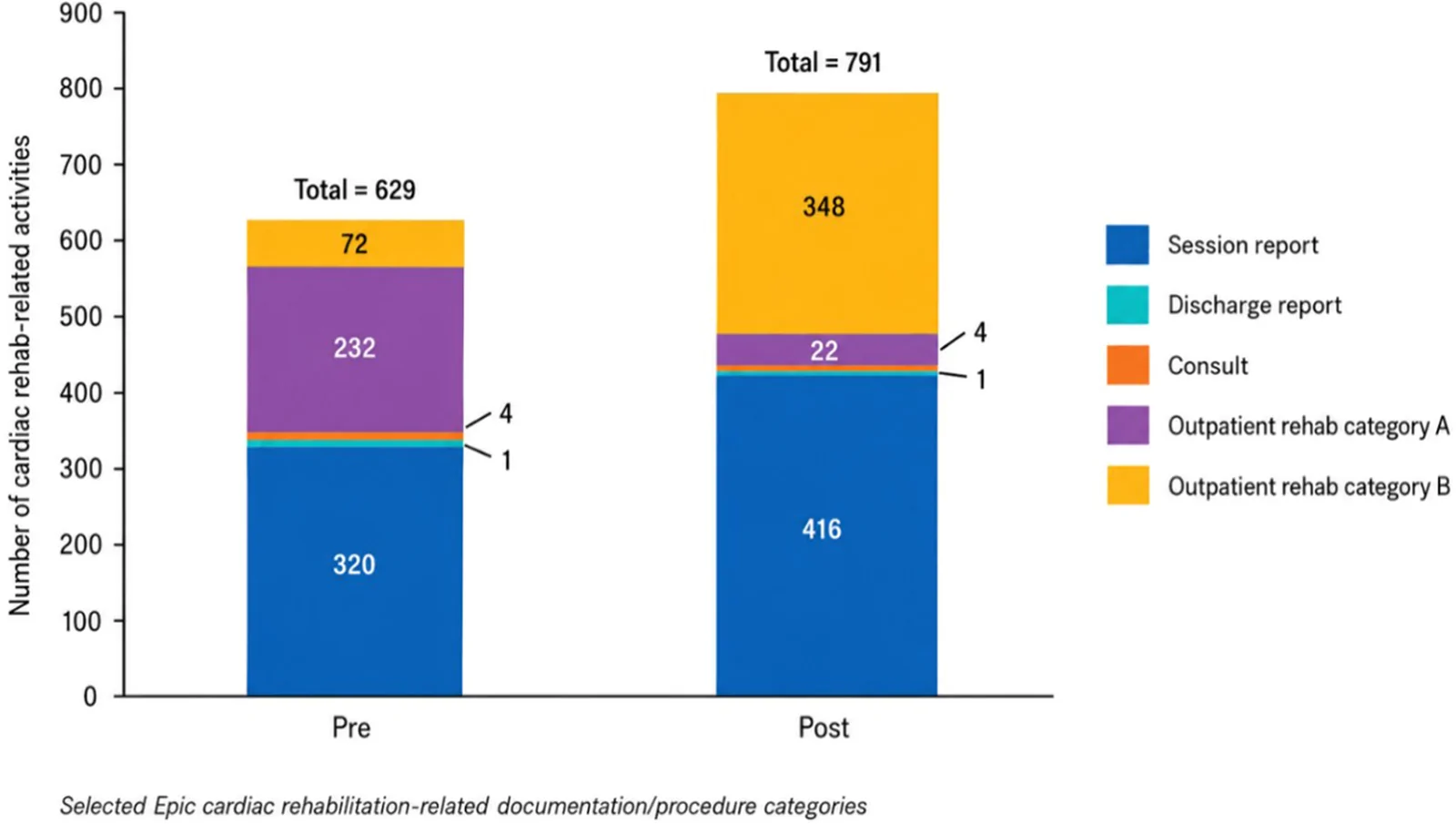
Composition of Cardiac Rehabilitation Referral Orders and Related Documentation Activity

## Operational Impact

Providers were able to acknowledge the BPA; however, completion of the referral workflow depended on provider action. Providers also had the option to close the BPA without placing a referral order, provided they documented a clinician-selected reason for not placing the referral. Although documentation of the referral-related activity improved, some alerts were acknowledged without placement of a referral order, suggesting opportunities to improve workflow design and provider engagement to streamline and standardize cardiac rehabilitation referral processes within the discharge pathway.

## Discussion

This QI initiative demonstrates that embedding clinical decision support tools within the EHR can improve adherence to guideline-recommended CR referral practices. The observed increase in referral rates suggests that structured prompts at the point of discharge can reduce variability in provider behavior and improve standardization of care.

These findings are consistent with prior literature demonstrating that electronic referral systems and structured workflows enhance CR use.^5^^,^^7^^,^^8^ However, the modest absolute improvement indicates that decision support alone may be insufficient to achieve optimal referral rates.

Previous studies evaluating automated CR referral pathways have demonstrated substantial improvements in referral rates, with reported referral rates ranging from approximately 52% to 86% depending on the degree of automation and integration with clinical workflows.^9^ Studies using electronic referral systems or automatic discharge-based referral pathways achieved referral rates around 70%, while combined automated referral with provider liaison approaches achieved rates approaching 86%. In contrast, usual referral processes have typically resulted in substantially lower referral rates, ranging from approximately 17% to 45%.

Compared with these prior studies, the improvement observed in our study may reflect the advantages of integrating an automated Epic-based BPA workflow directly into routine clinical practice, reducing reliance on individual provider recognition and manual referral processes.^9^^,^^10^

A key limitation identified in this project is incomplete provider engagement with BPA alerts, as many alerts were acknowledged without subsequent action. This finding highlights challenges related to alert fatigue, competing clinical priorities, and integration within existing discharge workflows.

Additional limitations include the observational pre-post study design, potential residual confounding, and the inability to evaluate whether patients who received referrals ultimately enrolled in or completed cardiac rehabilitation.

Future efforts should focus on refining order design, integrating automated referral pathways, and implementing structured order sets within the discharge workflow to further standardize and streamline CR referral processes and improve provider adherence.

## Conclusions

Implementation of an EHR-based clinical decision support tool increased CR referral rates among eligible cardiovascular patients. However, persistent gaps in provider engagement highlight the need for further optimization of workflow integration and decision support design. Refining these systems may improve adherence to guideline-directed cardiovascular care and support better long-term outcomes. Future studies should evaluate whether improved referral translates into increased CR enrollment and improved patient outcomes.

**Take-Home Messages**

• Electronic health record–based clinical decision support increased cardiac rehabilitation referral rates among eligible hospitalized patients.
• Provider engagement with electronic prompts remains inconsistent, limiting the full potential of the intervention.
• Optimizing integration of decision support into clinical workflows is essential to improve adherence to guideline-directed cardiovascular care.

## Funding Support and Author Disclosures

The authors have reported that they have no relationships relevant to the contents of this paper to disclose.
